# Supplementary material for: Plastic pollution fosters more microbial growth in lakes than natural organic matter
Source: Nat Commun. 2022 Jul 26;13:4175. doi: 10.1038/s41467-022-31691-9 (PMC9325981; doi:10.1038/s41467-022-31691-9)
Supplement: Supplementary file 1 — Supplementary Information [file 41467_2022_31691_MOESM1_ESM.docx]

**Supplementary Information for Plastic pollution fosters more microbial growth in lakes than natural organic matter**

Eleanor A. Sheridan, Jérémy A. Fonvielle, Samuel Cottingham, Yi Zhang, Thorsten Dittmar, David C. Aldridge, Andrew J. Tanentzap

**Supplementary Methods 1. Deriving environmentally relevant leachate concentrations**

To calculate an environmentally relevant DOC concentration released by plastic leachate, we first took the concentration of plastics found on the beaches of lakes in a highly populated area of southern Europe. The median value for macro- and micro-plastic concentrations combined was **0.59 g m^-2^** ^1^. The median was taken over the mean due to the high standard deviation indicating the presence of outliers that would bias the latter upwards. This value was then converted to volume based on the density of LDPE – the most commonly found plastic in lakes ^2^. The mean density of LDPE is **0.925 g L^-1^** ^3^, making the median volume of plastic 0.59 g m^-2^ / 0.925 g L^-1^ = **0.638 cm^3^ m^-2^**. We then assumed that plastic pollution in our lakes was predominately derived from plastic shopping bags ^2^, and converted the volume to an area basis. Assuming plastic bags are 0.0025 cm thick ^4^, 1 cm^3^ of plastic bag = **4000 cm^2^ cm^-3^**. Therefore, the median lake plastic volume will be 0.638 cm^3^ m^-2^ × 4000 cm^2^ cm^-3^ = **2552 cm^2^ m^-2^**. Since LDPE is buoyant in freshwaters ^3^, we assumed that most plastic will be concentrated in the top 15 cm × 1.0 m^2^ = **150 L** of surface water. Surface plastic concentration of lake water would thus be 2552 cm^2^ m^-2^ / 150 L m^-2^ = **17.01 cm^2^** **L^-1^**. Finally, we used this value to estimate how much carbon would leach from plastic in surface waters. Romera-Castillo et al. (2018) found that the leaching rate of DOC from plastic bags under idealised laboratory conditions was **6.67 μg C cm^-2^**. Therefore, the plastic in lakes may leach 17.01 cm^2^ L^-1^ × 6.67 μg C cm^-2^ = 113 μg C L^-1^ ≅ **0.1 mg C L^-1^**. Therefore, plastic leachate was added to our experiment at a final concentration of **0.1 mg L^-1^**.

**Supplementary Figure 1. The 29 lakes (dark blue circles) sampled across Sweden, Norway, and Finland between 6^th^ August and 23^rd^ September 2019.** Maps made with Natural Earth (https://www.naturalearthdata.com/about/terms-of-use/)

**Supplementary Figure 2.** **Flowchart showing incubation treatments and sample processing**. Yellow boxes indicate measurements taken, red boxes indicate the addition of plastic leachate or a control addition of distilled water, and blue boxes indicate lake water.

**Supplementary Figure 3.** **Labile molecules are relatively more abundant with both lower DOC concentration and FD**. We calculated (a) DOC and (b) FD in waters of 22 lakes after we added plastic leachate. We corrected for the dilution of molecular formulas in plastic leachate by multiplying the peak intensity of each formula by the proportion of plastic-derived DOC that was added to existing lake DOC before summing the intensities of shared molecular formulas in the mixture. We then summed the relative intensity of only those molecules classed as having a high lability index based on a H:C ratio ≥1.5 after D’Andrilli et al.^5^ and performed regression analysis.

**
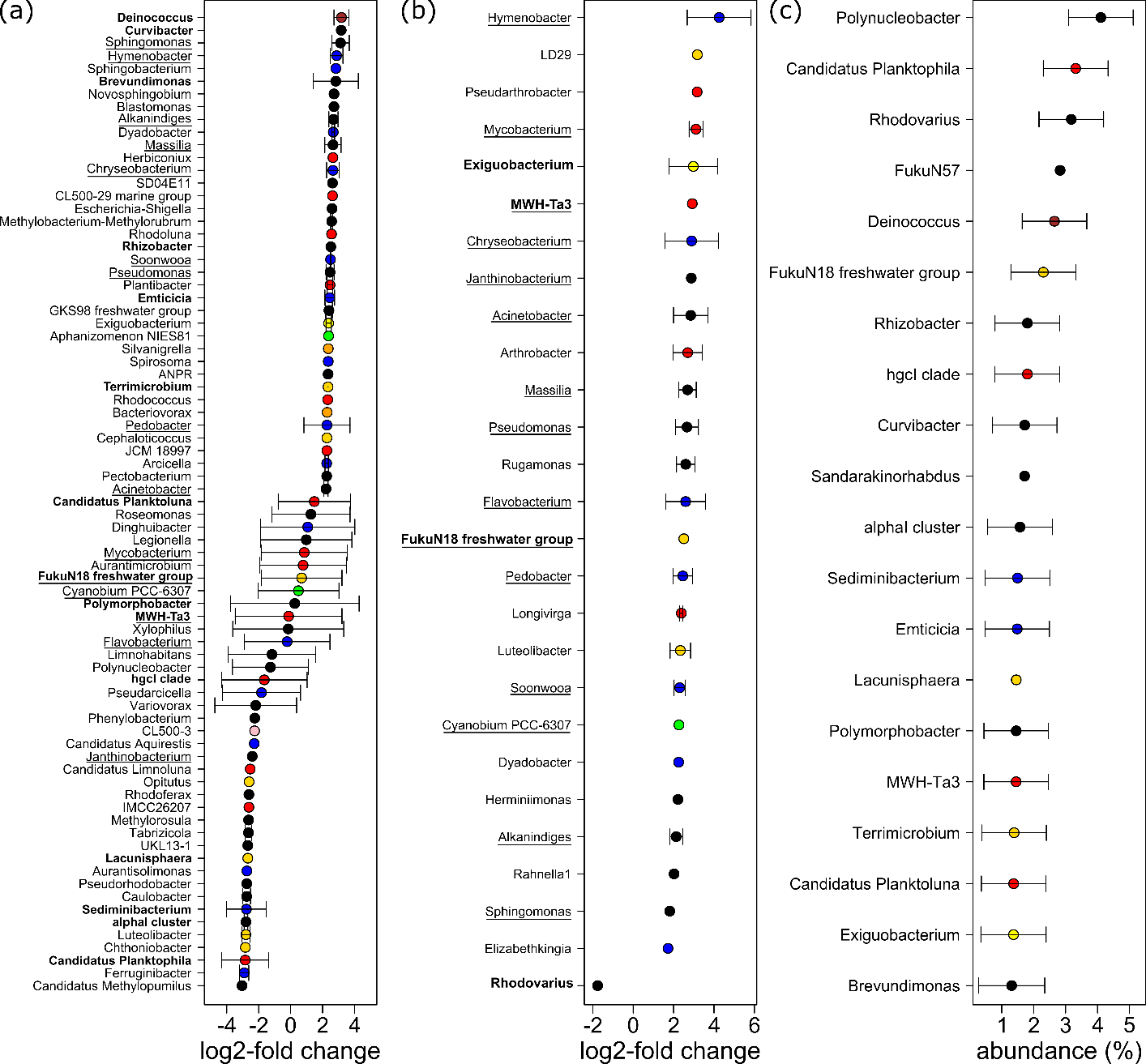
**

**Supplementary Figure 4**. **Use of plastic leachate positively correlates with abundances of different microbial taxa**. Each dot is the mean log2-fold change in normalized abundance of a given genus with increasing fold change in (**a**) BGE or (**b**) BPP. Responses of individual ASVs were averaged at the genus-level, n = 540 and 154 ASVs for (**a**) and (**b**), respectively. (**c**) Mean relative abundance (percent of total reads) in each lake for the 20 most abundant genera. For all, error bars are the standard deviation when more than one ASV was included in a genus. Underlined genera were always associated with community-level metabolism, that is, occurred in (**a**) and (**b**). Bold genera were the most abundant and so occurred in (**c**) and either (**a**) or (**b**). Colors denote phyla: Actinobacteriota (red), Bacteroidota (blue), Bdellovibrionota (orange), Cyanobacteria (green), Deinococcota (brown), Firmicutes (yellow), Planctomycetota (pink), Proteobacteria (black), and Verrucomicrobiota (gold). ANPR = *Allorhizobium*-*Neorhizobium*-*Pararhizobium*-*Rhizobium*.

**Supplementary References:**

1. Faure, F., Demars, C., Wieser, O., Kunz, M. & de Alencastro, L. F. Plastic pollution in Swiss surface waters: nature and concentrations, interaction with pollutants. *Environ. Chem.* **12**, 582 (2015).

2. Andrady, A. L. Microplastics in the marine environment. *Marine Pollution Bulletin* **62**, 1596–1605 (2011).

3. Bond, T., Ferrandiz-Mas, V., Felipe-Sotelo, M. & Sebille, E. van. The occurrence and degradation of aquatic plastic litter based on polymer physicochemical properties: A review. *Critical Reviews in Environmental Science and Technology* **48**, 685–722 (2018).

4. The Thick and Thin of Plastic Bags. *Multi-Pak USA Inc.* https://multipakusa.com/blogs/news/the-thick-and-thin-of-plastic-bags (2017).

5. D’Andrilli, J., Cooper, W. T., Foreman, C. M. & Marshall, A. G. An ultrahigh-resolution mass spectrometry index to estimate natural organic matter lability. *Rapid Communications in Mass Spectrometry* **29**, 2385–2401 (2015).
