# Supplementary material for: Plastic pollution fosters more microbial growth in lakes than natural organic matter
Source: Nat Commun. 2022 Jul 26;13:4175. doi: 10.1038/s41467-022-31691-9 (PMC9325981; doi:10.1038/s41467-022-31691-9)
Supplement: Supplementary file 3 — Reporting Summary [file 41467_2022_31691_MOESM3_ESM.pdf]

nature portfolio

Corresponding author(s): Andrew Tanentzap and Eleanor Sheridan

Last updated by author(s): May 4, 2022

# Reporting Summary

Nature Portfolio wishes to improve the reproducibility of the work that we publish. This form provides structure for consistency and transparency in reporting. For further information on Nature Portfolio policies, see our Editorial Policies and the Editorial Policy Checklist.

## Statistics

For all statistical analyses, confirm that the following items are present in the figure legend, table legend, main text, or Methods section.

| n/a | Confirmed | |
|---|---|---|
| ☐ | ☒ | The exact sample size ($n$) for each experimental group/condition, given as a discrete number and unit of measurement |
| ☐ | ☒ | A statement on whether measurements were taken from distinct samples or whether the same sample was measured repeatedly |
| ☐ | ☒ | The statistical test(s) used AND whether they are one- or two-sided<br>*Only common tests should be described solely by name; describe more complex techniques in the Methods section.* |
| ☐ | ☒ | A description of all covariates tested |
| ☐ | ☒ | A description of any assumptions or corrections, such as tests of normality and adjustment for multiple comparisons |
| ☐ | ☒ | A full description of the statistical parameters including central tendency (e.g. means) or other basic estimates (e.g. regression coefficient) AND variation (e.g. standard deviation) or associated estimates of uncertainty (e.g. confidence intervals) |
| ☐ | ☒ | For null hypothesis testing, the test statistic (e.g. $F$, $t$, $r$) with confidence intervals, effect sizes, degrees of freedom and $P$ value noted<br>*Give P values as exact values whenever suitable.* |
| ☒ | ☐ | For Bayesian analysis, information on the choice of priors and Markov chain Monte Carlo settings |
| ☒ | ☐ | For hierarchical and complex designs, identification of the appropriate level for tests and full reporting of outcomes |
| ☐ | ☒ | Estimates of effect sizes (e.g. Cohen's $d$, Pearson's $r$), indicating how they were calculated |

*Our web collection on statistics for biologists contains articles on many of the points above.*

## Software and code

Policy information about availability of computer code

| | |
|---|---|
| Data collection | ICBM-OCEAN was used to assign molecular formulas to DOM. DADA2 was used to assign taxonomy to 16S rRNA amplicons. |
| Data analysis | All data was analysed in R version 3.5.3 |

For manuscripts utilizing custom algorithms or software that are central to the research but not yet described in published literature, software must be made available to editors and reviewers. We strongly encourage code deposition in a community repository (e.g. GitHub). See the Nature Portfolio guidelines for submitting code & software for further information.

## Data

Policy information about availability of data

All manuscripts must include a data availability statement. This statement should provide the following information, where applicable:

- Accession codes, unique identifiers, or web links for publicly available datasets
- A description of any restrictions on data availability
- For clinical datasets or third party data, please ensure that the statement adheres to our policy

The BPP and BGE data generated in this study can be downloaded from FigShare (https://figshare.com/articles/dataset/BPP_data/19692031; https://figshare.com/articles/dataset/BGE_data/19692028). The DNA sequences can be downloaded from the EBI database (https://www.ebi.ac.uk/services/dna-rna) under accession number PRJEB49321 https://www.ebi.ac.uk/ena/browser/view/PRJEB49321.

# Field-specific reporting

Please select the one below that is the best fit for your research. If you are not sure, read the appropriate sections before making your selection.

☐ Life sciences ☐ Behavioural & social sciences ☒ Ecological, evolutionary & environmental sciences

For a reference copy of the document with all sections, see nature.com/documents/nr-reporting-summary-flat.pdf

# Ecological, evolutionary & environmental sciences study design

All studies must disclose on these points even when the disclosure is negative.

| | |
|---|---|
| Study description | 29 lakes were sampled<br>3 replicates were taken for each treatment (plastic leachate addition, distilled water addition, no addition (for time 0 processing) for BPP<br>2 replicates were taken for each treatment (plastic leachate addition, distilled water addition, no addition (for time 0 processing) for respiration<br>3 replicates were taken for DOC concentration<br>1 replicate was taken for functional diversity<br>1 replicate was take for microbial community composition estimated from 16S rRNA amplicon sequencing |
| Research sample | 29 lakes were selected across Scandinavia to capture the range of environment conditions present in the region. |
| Sampling strategy | The sample size of 29 lakes was chosen to give a wide range of environmental conditions. |
| Data collection | ES and SC collected BPP data using a Triathler liquid scintillation counter<br>ES collected respiration data using fiber-optics optodes connected to a OXY-1 ST meter<br>JF collected DOC concentration, DOM functional diversity, and microbial community composition data |
| Timing and spatial scale | Lakes were located in Sweden, Norway, and Finland between latitudes of 59.1N and 70.3N<br>The first lake was sampled on 10th August 2019 and the final lake sampled on 19th September 2019 |
| Data exclusions | 11 lakes were excluded from BGE analysis due to not possessing respiration data.<br>7 lakes were excluded from the DOM analysis due to samples not being taken.<br>9 lakes were excluded from the microbial community analysis due to samples not being taken. |
| Reproducibility | Replicates were taken to ensure reproducibility: 3 for each BPP treatment, 2 for each respiration treatment, 3 for DOC concentration |
| Randomization | Not relevant to our study |
| Blinding | Not relevant to our study |

Did the study involve field work? ☒ Yes ☐ No

## Field work, collection and transport

| | |
|---|---|
| Field conditions | Field conditions were consistent with expected weather for the location and time of year. Temperatures ranged from approx 0C to 30C |
| Location | Lakes were located in Sweden, Norway, and Finland between latitudes of 59.1N and 70.3N |
| Access & import/export | All lakes were publicly accessible and no permits were required. |
| Disturbance | We caused no disturbance to lakes |

# Reporting for specific materials, systems and methods

We require information from authors about some types of materials, experimental systems and methods used in many studies. Here, indicate whether each material, system or method listed is relevant to your study. If you are not sure if a list item applies to your research, read the appropriate section before selecting a response.

## Materials & experimental systems

| n/a | Involved in the study |
| --- | --- |
| ☒ | ☐ Antibodies |
| ☒ | ☐ Eukaryotic cell lines |
| ☒ | ☐ Palaeontology and archaeology |
| ☒ | ☐ Animals and other organisms |
| ☒ | ☐ Human research participants |
| ☒ | ☐ Clinical data |
| ☒ | ☐ Dual use research of concern |

## Methods

| n/a | Involved in the study |
| --- | --- |
| ☒ | ☐ ChIP-seq |
| ☒ | ☐ Flow cytometry |
| ☒ | ☐ MRI-based neuroimaging |
